# Antiviral and Anti-Inflammatory Plant-Derived Bioactive Compounds and Their Potential Use in the Treatment of COVID-19-Related Pathologies

**DOI:** 10.3390/jox12040020

**Published:** 2022-10-08

**Authors:** Purvi Trivedi, Amna Abbas, Christian Lehmann, H. P. Vasantha Rupasinghe

**Affiliations:** 1 Department of Anesthesia, Pain Management and Perioperative Medicine, Faculty of Medicine, Dalhousie University, Halifax, NS B3H 3E2, Canada; 2 Department of Plant, Food, and Environmental Sciences, Faculty of Agriculture, Dalhousie University, Truro, NS B2N 5E3, Canada; 3 Department of Biochemistry and Biomedical Sciences, McMaster University, Hamilton, ON L8S 4M1, Canada; 4 Department of Pathology, Faculty of Medicine, Dalhousie University, Halifax, NS B3H 3E2, Canada

**Keywords:** coronavirus, SARS-CoV-2, viral infections, phytochemicals, respiratory tract, cytokine storm, inflammation

## Abstract

The highly contagious coronavirus disease (COVID-19), caused by severe acute respiratory syndrome coronavirus 2 (SARS-CoV-2), has been declared a global pandemic and public health emergency as it has taken the lives of over 5.7 million in more than 180 different countries. This disease is characterized by respiratory tract symptoms, such as dry cough and shortness of breath, as well as other symptoms, including fever, chills, and fatigue. COVID-19 is also characterized by the excessive release of cytokines causing inflammatory injury to the lungs and other organs. It is advised to undergo precautionary measures, such as vaccination, social distancing, use of masks, hygiene, and a healthy diet. This review is aimed at summarizing the pathophysiology of COVID-19 and potential biologically active compounds (bioactive) found in plants and plant food. We conclude that many plant food bioactive compounds exhibit antiviral and anti-inflammatory properties and support in attenuating organ damage due to reduced cytokine release and improving the recovery process from COVID-19 infection.

## 1. Introduction

Coronavirus disease 2019 (COVID-19) emerged in the city of Wuhan, China, in December 2019 and is currently a global pandemic. COVID-19 is caused by severe acute respiratory syndrome coronavirus 2 (SARS-CoV-2) and is an ongoing global health emergency. As per the World Health Organization, SARS-CoV-2 has been confirmed in nearly 400 million people worldwide, and over 5.7 million people have died due to the illness [1]. Among the 2.5% of people who have died from COVID-19, 74% of them are over 65 years of age, with age representing a major risk factor for death in COVID-19 patients [1]. Furthermore, people who are immunocompromised or present with several comorbidities, such as diabetes, respiratory diseases, hypertension, obesity, cancer, and cardiovascular complications, are also at higher risk of contracting COVID-19 [2]. SARS-CoV-2 is closely related to SARS-CoV and Middle East respiratory syndrome (MERS) and infects the lower respiratory tract, resulting in acute lung injury (ALI)/acute respiratory distress syndrome (ARDS), eventually leading to pneumonia with fever, cough, and dyspnea [3]. About 80% of SARS-CoV-2-positive patients exhibit mild symptoms or are asymptomatic, whereas the remaining 20% of patients develop more severe symptoms, which can lead to ARDS, septic shock, multiple organ failure, and death. Human-to-human transmission occurs through close contact with an infected person, aerosol spread, viral shedding by asymptomatic people, airborne transmission, and during medical procedures. Viral shedding can also occur from the respiratory tract, coughing, sneezing, and saliva, leading to virus spread [4,5,6]. 

Although vaccines have been developed against SARS-CoV-2, they can lose their efficacy if the virus mutates and alters its antigenicity so that it no longer closely matches the antigen used in vaccine production. Until now, no drugs have been approved as a complete cure for SARS-CoV-2 infection. Possible therapies can be categorized into: 1. drugs targeting the virus and 2. drugs acting on human cells, e.g., the immune system. Viral targets consist of both nonstructural proteins and structural proteins; however, if the virus mutates, these therapies may also become ineffective. Therefore, drugs targeting host-cell viral receptors and modulation of immune system and inflammation have strong potential. The US Food and Drug Administration (FDA) has temporarily approved antiviral and anti-inflammatory medications that are still experimental and are only given to critically ill patients. Furthermore, many current treatments used by physicians only manage symptoms, such as breathing support using mechanical ventilation and corticosteroid administration to control lung swelling [7,8]. Hence, there is a need for safe and effective medications to prevent the progression of the illness at any stage. 

Various naturally occurring, plant-derived bioactive molecules have well-described antioxidative, anti-inflammatory, and immunomodulatory properties and could likely be used as potential therapies for COVID-19. Natural remedies have long been used to treat inflammatory and infectious diseases. In one study, traditional Chinese medicine significantly reduced both the length of stay in the hospital and corticosteroid-mediated side effects in patients suffering from SARS infection in 2003 [9]. The majority of anti-inflammatory, antiviral, and immunomodulatory effects of plant-derived bioactive compounds are described for the treatment of HIV [10], hepatitis [11], influenza [12], or herpes simplex virus type 1 (HSV-1) [13]. In this review, we have explored the anti-inflammatory, anti-viral and immunomodulatory effects of plant-derived bioactive compounds and the utility of using phytochemicals as a treatment option for SARS-CoV-2 infection. We have reviewed in vivo, in vitro, and clinical trial studies on plant-derived bioactive compounds, such as polyphenols, alkaloids, cannabinoids, plant lipids, and prebiotic polysaccharides, and the possibility of applying findings to treat symptoms in patients infected with SARS-CoV-2.

## 2. COVID-19 Pathophysiology

### 2.1. Genome, Protein Structure, and Life Cycle

**Figure 1:**
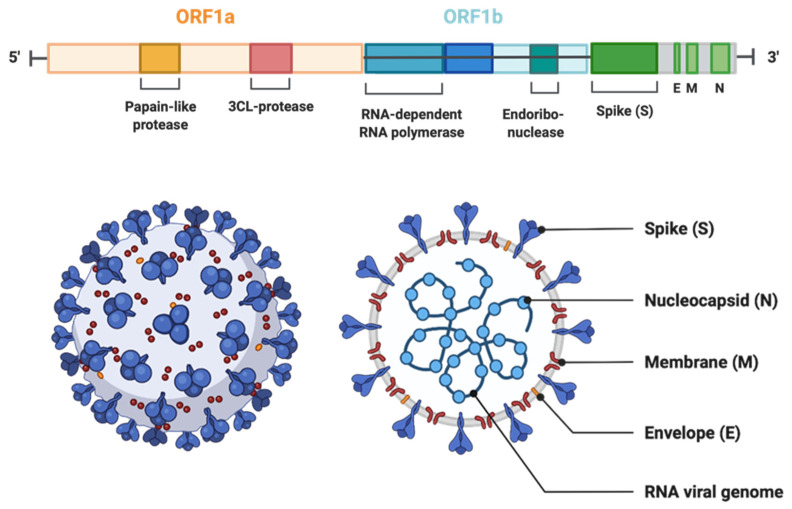
The genomic organization of SARS-CoV-2. The genome encodes two large genes ORF1 (yellow) and ORF1b (blue), which encode nonstructural proteins. These nonstructural proteins encode for papain-like protease (PLP), 3CL-protease, RNA-dependent RNA polymerase, and endoribonuclease. The structural genes encode the structural proteins, spike (S), envelope (E), membrane (M), and nucleocapsid (N) (highlighted in green). (The Figure is created with Biorender.com.)

**Figure 2:**
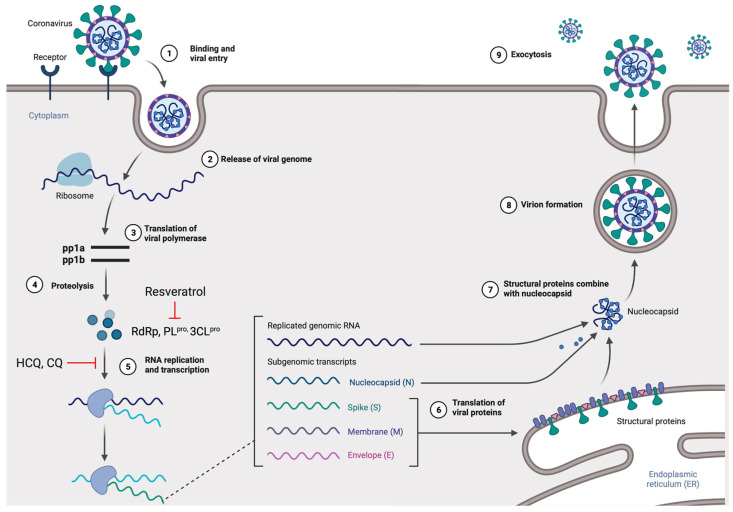
The life cycle of SARS-CoV-2 in the host cells. The S glycoprotein of the virion binds to the cellular receptor angiotensin-converting enzyme (ACE2) and enters the target cell through an endosomal pathway. Following the entry, the viral RNA is translated to produce pp1a and pp1ab, which are then cleaved by the proteases (RdRp, PL^pro^, and 3CL^pro^). During the RNA replication and transcription, genomic RNA and structural proteins (N, M, E, and S) were produced. Following the production of SARS-CoV-2 structural proteins, nucleocapsids are assembled in the cytoplasm and followed by budding into the lumen of the endoplasmic reticulum (ER)–Golgi intermediate compartments. Virions are then released from the infected cell through exocytosis. Red lines indicate possible antiviral mechanisms of bioactive compounds (figure created with Biorender.com).

Viruses in the *Coronaviridae* family, also known as corona or crown viruses, consist of unique structures of protruding peplomers, which are the so-called “crown”. The *Coronaviridae* family of viruses belong to the order *Nidavirales.* Coronaviruses are enveloped, positive single-stranded RNA viruses and have the largest known RNA viral genome of 8.4–12 kDa in size [14]. The International Committee on Taxonomy of Viruses (ICTV) categorizes the coronaviruses into four types: α-, β-, δ-, and γ–coronaviruses [15]. Both α- and β-CoVs infect mammals, while γ–CoVs infect avian species, and δ-CoVs infect both mammals and avian species. β-CoVs consist of SARS-CoV, mouse hepatitis CoV (MHV), MERS-CoV, bovine CoV (BCoV), bat CoV (HKU4), and human CoV (OC43 including SARS-CoV) [16] (Figure 1). The three CoVs, SARS-, MERS-, and SARS-CoV2 are spread through close contact and have zoonotic transmission [17]. The viral genome can be subdivided into the 5’ and 3’ terminals. The 5’ terminal constitutes a major portion of the genome and contains open reading frames (ORFs), which encode for proteins responsible for viral replication, such as RNA-dependent RNA polymerase (RdRp), papain-like protease (PL^pro^), and 3-chymotrypsin-like protease (3CL^pro^) [18,19]. In contrast, the 3’ end of the viral genome encodes for structural proteins, including nucleocapsid protein (N), envelope protein (E), membrane protein (M), spike protein (S), and haemagglutinin-esterase protein (HE) [18]. Among the structural proteins, viral surface protein S is extensively glycosylated and mediates attachment and fusion between the virus and the host cell membrane. The N protein forms RNA complexes that promote virus transcription and assembly. The M protein is the most abundant structural protein that defines the viral envelope shape, whereas the E protein is the smallest of all the structural proteins that is highly expressed in infected cells during the viral replication cycle. Lastly, the HE protein is responsible for receptor binding and host specificity [20,21]. Entry of SARS-CoV-2 is mediated by a cell surface receptor called angiotensin-converting enzyme-2 (ACE2). ACE2 receptor binds to receptor-binding domain (RBD) on SARS-CoV-2 spike protein. Following the RBD–receptor interaction, the S protein undergoes proteolytic cleavage by several host proteases, such as transmembrane serine protease 2 (TMPRSS2), cathepsin B or L (CTS-B or -L), and furain. Proteolytic processing of the S protein allows the virus to fuse with host cell membrane and discharge its viral RNA into the host cytoplasm. Subsequently, viral RNA utilizes the host and its machinery to replicate its genetic material to assemble new viral particles in the host cell cytoplasm [19,20,22] (Figure 2).

### 2.2. Potential Mechanism-Based Treatment of COVID-19 Infection

**Figure 3:**
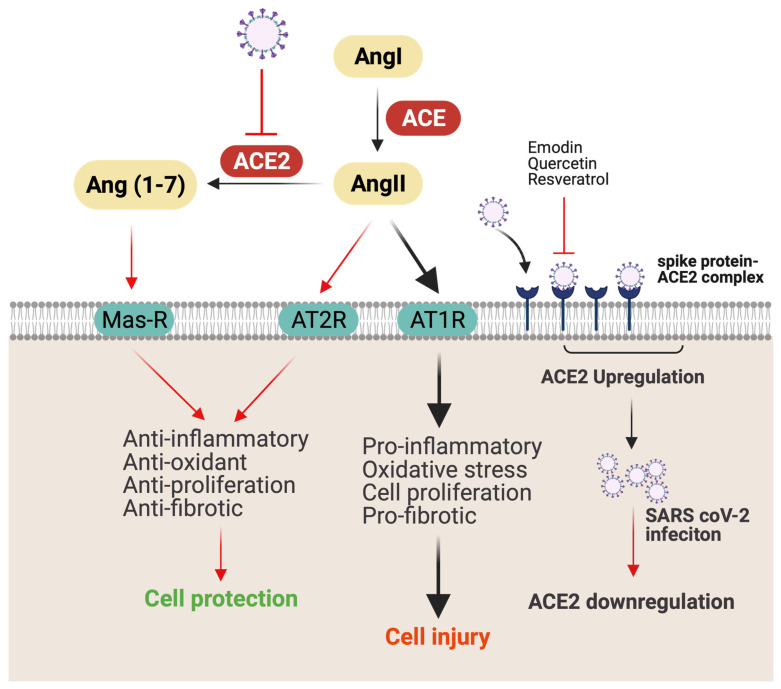
Possible mechanism of SARS-CoV-2 infection and lung injury. ACE converts AngI to AngII, which causes lung injury by promoting inflammation and fibrosis through AT1R. Meanwhile, by binding to AT2R, AngII causes opposite effects, such as anti-inflammatory, antioxidant, antiproliferation, and antifibrotic. ACE2 coverts AngII to Ang (1–7), which ameliorates inflammation by binding to Mas receptor (Mas-R). ACEe-binding SARS-CoV-2 is internalized by endocytosis, resulting in downregulation of membrane-anchored ACE2 on the cell surface. Downregulation of ACE2 decreases AngII conversation to Ang (1–7) and increases AngII binding with AT1R, causing cellular injury. Red line indicates possible antiviral and anti-inflammatory mechanisms of bioactive compounds (figure created with Biorender.com).

ACE2 receptors are found on various organs and tissues, including nasal mucosa, lung parenchyma, gastrointestinal (GI) and renal tract, vascular endothelium, reproductive system, and cerebral neurons, and act as a gateway for SARS-CoV-2 virus [23]. Consequently, ACE2 provides access to SARS-CoV-2 to different tissues, causing disorders of multiple organs, such as GI, heart, kidney, and lung [24]. Notably, ACE2 levels are downregulated following SARS-CoV-2 infection [24]. Decreased ACE2 expression reduces angiotensin-II (AngII) conversion to Ang (1–7), increases the secretion of the AngII, and reduces the secretion of vasodilator angiotensin (1–7) [25]. ACE2 receptor deficiency is associated with an increase in severity of acute lung injury, respiratory distress syndrome, lung edema, and neutrophil accumulation [23]. Additionally, AngII plays an important role in mediating proinflammatory response through angiotensin receptor 1 (AT1R). Increased secretion of AngII results in the activation of NF-kB, which further increases the expression of EFGR and TNFα (Figure 3) [26]. Notably, higher levels of ACE2 receptors in lung epithelial cells in children and young adults appears to have a protective effect on COVID-19 clinical manifestations [27]. Therefore, therapy to upregulate ACE2 protein expression may provide protection against AngII-mediated detrimental effects.

In addition to ACE2 receptors, 3-chymotrypsin-like protease (3CLpro) and S spike glycoprotein are also identified as attractive targets to combat COVID-19 disease. 3CLpro protease not only cleaves polyproteins to viral-related proteins, which is crucial for virus replication and maturation, but also cleaves NF-kB transcription factor, which is essential for regulating cell survival and immune response [28]. Meanwhile, S spike glycoprotein and TMPRSS2 protease play an important role of viral entry to host cell by binding to ACE2 receptor to assist viral entry to host cells.

**Figure 4:**
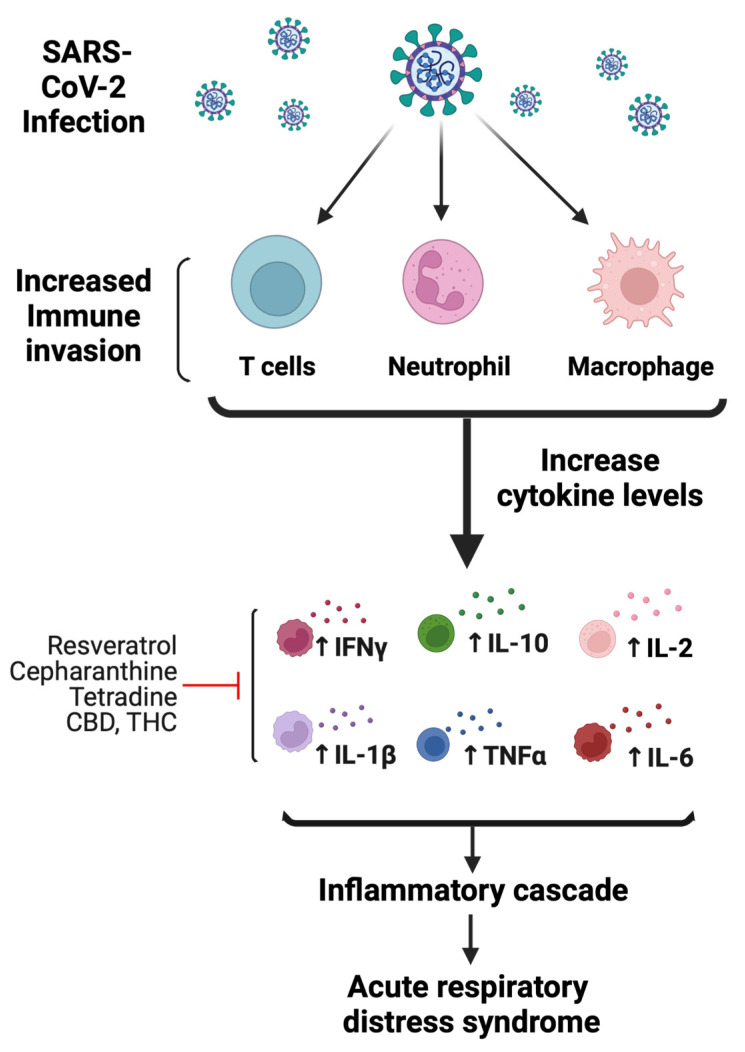
Mechanisms of SARS-CoV-2-mediated immunomodulatory and inflammatory response. Following infection, SARS-CoV-2 hyperactivates immune cells, such as T cells, macrophages, and neutrophils. Initial increase in immune response activates proinflammatory cytokines. As a result, cytokine levels increase, which include IL-2, IL-6, IL-7, IL-10, IFN-γ, and TNFα. The increase in immune response leading to increased cytokine levels is associated with lung pathology likely causing acute respiratory distress syndrome. Red line indicates the possible anti-inflammatory mechanism of bioactive compounds (figure created with Biorender.com).

Dysregulation of the host immune response and activation of inflammatory cytokines are common events following viral infection [29]. Following infection, SARS-CoV-2 induces a noneffective host immune response leading to increased cytokine levels that are associated with lung pathology and eventually death. The release of these high levels of cytokines is referred to as a “cytokine storm”, which is characterized by increased plasma concentrations of granulocyte-colony-stimulating factor (GCSF), IL-2, IL-6, IL-7, IL-10, IFN-γ inducible protein 10 (IP-10), macrophage inflammatory protein 1-α, and TNFα [30,31] (Figure 4). The immune response initially consists of an adaptive immune response necessary to control virus replication and to prevent disease progression. However, once the virus enters lung tissue, there is a compensatory upregulation of the general inflammatory response to combat the viral infection. This host inflammatory response is driven by binding to toll-like receptors (TLRs), which recognize structural components belonging to viruses, known as “pathogen-associated molecular patterns” (PAMPs). The continuous release of cytokines hyperactivates immune cells, such as T cells, macrophages, dendritic, and endothelial cells, which further release more cytokines (Figure 4). Furthermore, cytokine storm is also enhanced by unbalanced levels of AngII/Ang (1–7), which could negatively affect the cardiovascular system in COVID-19-infected patients.

### 2.3. Treatment Options for COVID-19 Patients

Although vaccines have been approved for the prevention of COVID-19, there is still an urgent need for efficacious drugs to treat COVID-19. Pharmaceutical companies and laboratories worldwide are working to develop medicines for COVID-19 and many clinical trials are underway to test drugs. Depending on the severity of the infection, the treatment for COVID-19 varies. For milder illness, resting at home and taking medication to reduce fever is often sufficient, whereas more severe illness requires hospitalization and treatment with supplementation of oxygen and assisted ventilation. The few drugs which have received full approval from FDA to treat COVID-19 patients include the antiviral drug (remdesivir (Veklury™)), 3CLpro protease inhibitor (Nirmatrelvir/ritonavir (Paxlovid)) [32], IL-6 inhibitors (sarilumab, tocilizumab, and siltuximab), and the corticosteroid dexamethasone. Remdesivir was initially developed to treat Ebola virus infection. The US clinical trial ACTT-1 suggested a 31% shorter recovery time (10 days vs. 15 days) in COVID-19 patients treated with remdesivir compared to patients who did not receive the drug [33]. Dexamethasone has been used for many years to treat inflammation during asthma, Crohn’s disease, and certain cancer. The RECOVERY trial (NCT04381936) studied the steroid drug dexamethasone to treat inflammation and reduce deaths in hospitalized COVID-19 patients. IL-6 inhibitors have been evaluated in COVID-19 patients to manage systemic inflammation. In some COVID-19 patients who are exhibiting rapid respiratory decompensation, tocilizumab is given in combination with dexamethasone. Lastly, the FDA has also given emergency authorization to use convalescent plasma (CP) in hospitalized patients; however, the usefulness of CP is less robust than that for remdesivir or dexamethasone (NCT04381936). It is important to note that these drugs are only directed to relieve COVID-19-related symptoms. No antiviral drug has been approved to treat COVID-19 specifically. Alternatively, plant-derived bioactive compounds have been used for thousands of years to treat various viral-related illnesses and could likely be used as valuable resources to treat symptoms in COVID-19 patients.

## 3. Evidence for Potential Benefits of Plant-Derived Bioactives

A regulated immune response is a hallmark of physiology, which defends the human body towards infections due to microorganism invasion and other internal and external insults. A suitable nutrient and dietary bioactive supply are necessary to strengthen the immune response through all phases of life. Plant-derived bioactive compounds are effective in mitigating infections by boosting immune response through a variety of mechanisms. In the following section, the properties of bioactive compounds and their potential impact on SARS-CoV-2 infection will be discussed.

### 3.1. Polyphenols

**Figure 5:**
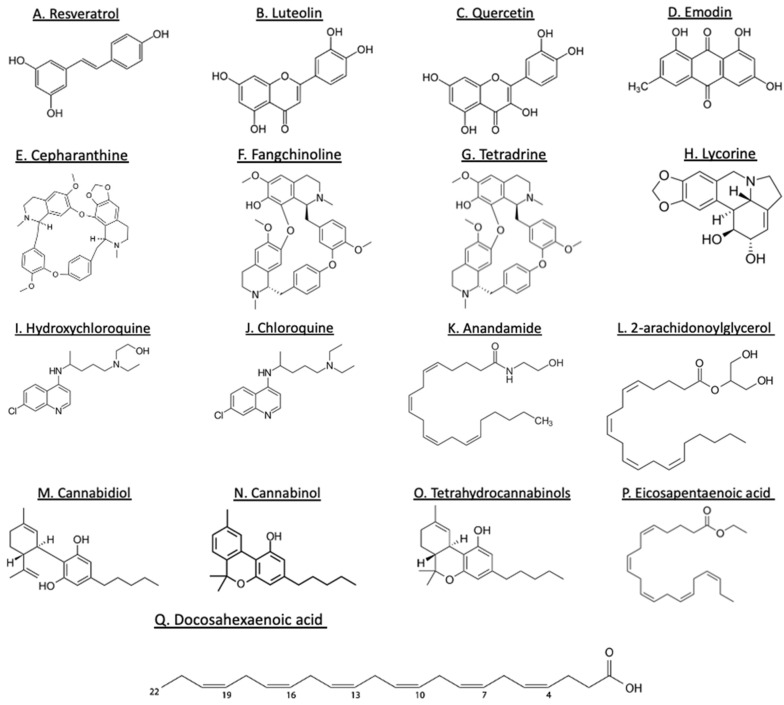
Structures of some plant-derived bioactive compounds. Polyphenols (**A**–**D**), alkaloids (**E**–**J**), cannabinoids (**K**–**O**), and lipids (**P**–**Q**).

Plant-derived polyphenols have various bioactive phenolic compounds that have the potential to prevent the development of several diseases [34,35]. Polyphenol constituents are categorized on the basis of their molecular mass, chemical construction, and intricacy to flavonoids (flavones, flavonols, flavanones, flavanonols, isoflavonoids, flavanols, and anthocyanidins) and nonflavonoid (phenolic acids, stilbenes, curcuminoids, lignans, tannins, and others) (Figure 5). These polyphenols are isolated from a variety of plants, such as fruits, vegetables, legumes, nuts, seeds, and herbs [36]. Polyphenol components have gained considerable attention for their biological properties, such as antiviral, antibacterial, antioxidative, and anti-inflammatory effects [37]. Although the consumption of polyphenols does not guarantee a consistent antiviral effect, a large number of in vitro and in vivo studies have demonstrated the immunomodulatory effect of polyphenols by regulating proinflammatory gene expression and cytokine production [38]. Resveratrol, which is found abundantly in the skins of red grapes, wine, peanuts, cocoa, and berries [39], attenuates oxidative stress and inflammation in lung fibroblasts [40] and the mouse kidney [41]. Furthermore, the consumption of resveratrol has been proven to be beneficial during obesity due to its powerful aid in controlling the inflammatory response, such as preventing the activation of NLRP3 inflammasome, reducing IL-1, IL-6, and TNF-α production in the liver and adipose tissue of obese mice [18,40] (Table 1). Additionally, resveratrol, curcumin, and green tea polyphenols have also shown a protective role in zymosan-induced multiple-organ dysfunction syndrome models [42,43]. Together, these findings highlight the importance of bioactive polyphenols in different diseases by modulating inflammatory pathways, likely implying the beneficial role of polyphenols during SARS-CoV-2 infection.

In addition to their antioxidative and anti-inflammatory effects, polyphenols have also been tested for their anti-infective properties [44]. Numerous studies have demonstrated the beneficial effects of polyphenols against diverse families of viruses, such as influenza A virus (H1N1), hepatitis B and C viruses (HBV/HCV), herpes simplex virus 1 (HSV-1), human immunodeficiency virus (HIV), and Epstein–Barr virus (EBV) [45]. Polyphenols have the capacity to interrupt the life cycle of viruses, halt viral replication, and improve immune response. Flavonoids, luteolin, and quercetin have been shown to bind with S protein of SARS-CoV with high affinity and prevent SARS-CoV infection by inhibiting virus entry into Vero E6 cells, a cell line established from isolated kidney epithelial cells of an African green monkey [46], suggesting a possible anti-SARS-CoV-2 mechanism by targeting the S protein and viral entry.

Emodin, an anthraquinone-type polyphenol found in rhubarb roots, interferes with S protein-ACE2 interaction in a cell-free competition assay and decreases the infectivity of S protein-pseudotyped retrovirus to Vero E6 cells [47] (Table 1). Although the entry of SARS-CoV-2 into the host cell is mediated via ACE2 receptor, the level of ACE2 receptors is downregulated following the infection, which likely increases the ACE–AngII–AT1 receptor axis and causes AngII-mediated detrimental effects, such as hypertension, thrombosis, and inflammation [48]. Indeed, mice with ACE2 receptor knockout developed more severe acute lung injury and respiratory distress syndrome compared to wild-type mice following SARS-CoV-2 infection, whereas administration of recombinant ACE2 reversed the symptoms [23]. Importantly, dietary intake of polyphenol, specifically resveratrol, has the ability to upregulate ACE2 expression and activity [49,50,51]. Resveratrol treatment in obese mice increases ACE2 expression and inhibits the growth of abdominal aortic aneurysms [49]. Together, these studies indicate a potential for polyphenols to protect against severe lung injury associated with COVID-19 through modulation of ACE2 receptor expression.

SARS-CoV-2 viral proteases have been suggested to be effective targets to inhibit the virus life cycle. Viral proteases proteolytically process polyproteins into viral replication-related proteins, which is essential for viral replication and maturation. SARS-CoV-2 polyproteins are processed by 3CL^pro^ and PL^pro^. The flavonol aglycone quercetin suppresses 3CL^pro^ and inhibits a wide range of viruses, such as human T-lymphotropic virus 1, Japanese encephalitis virus, DENV-2, and HCV [52,53,54]. Another SARS-CoV-2 protease, RdRp, has proven to be a key target in the development of therapies against COVID-19. RdRp is an enzyme that catalyzes the replication of RNA from an RNA template and is an essential protein encoded in the genomes of all RNA-containing viruses, including SARS-CoV-2. In fact, remdesivir, an analog of adenosine, acts as a false substrate for RdRp and has been temporarily approved by the FDA for the treatment of COVID-19. Remdesivir incorporates into the viral RNA at position I, successfully inhibits RdRp, and terminates RNA synthesis, ultimately preventing viral proliferation [55].

Resveratrol significantly inhibits MERS-CoV replication in in vitro studies by inhibiting RNA expression and nucleocapsid protein expression [56]. Additionally, a molecular dynamic simulation study suggests a strong interaction of resveratrol with the S spike protein–ACE2 complex, likely inhibiting SARS-CoV2 viral replication [57]. Therefore, it is plausible that resveratrol may also be effective against SARS-CoV-2 infection by targeting both S spike protein–ACE2 complex formation and RdRp [58]. An ongoing phase 2 study aims to evaluate the effects of resveratrol in minimizing viral load and symptoms of COVID-19 infection (NCT04542993). A study of resveratrol with nutritional intervention is also underway to investigate its effect in reducing complications in patients with COVID-19 and comorbidities (NCT04507867). Furthermore, receptor binding and entry assay of 56 polyphenols in human alveolar epithelial cell line A549 revealed that curcumin has the highest binding affinity to the viral RBD of SARS-CoV-2 spike protein [59]. Molecular docking and molecular dynamic simulation studies also indicated that emodin blocks the interaction between SARS-CoV-2 S protein and ACE2 receptors [60]. Other polyphenols targeted against RdRp to treat COVID-19 were identified using a computational model. These polyphenols include fenoterol, a polyphenolic β-2 adrenergic receptor agonist, naturally occurring flavones, such as baicalin from *Scutellaria baicalensis,* and xanthenes from *Swerti apseudochinensis* [61]. These studies document anti-SARS-CoV-2 activity of polyphenols, providing scientific evidence for the future investigations in in vivo and clinical studies.

**Table 1:** Major findings in relation to the potential reduction of the impact of anti-COVID-19 by dietary plant food bioactive groups.

Plant Food Bioactive Group	Compound	Source	Observation	Reference
Polyphenols	Resveratrol Luteolin and quercetin Emodin	Grapes and berries Vegetables Rhubarb roots	- Attenuates oxidative stress and inflammation in lung fibroblast and mouse kidney - Decreases IL-1, IL-6, and TNF-α in mice liver and adipose tissue - Prevent SARS-CoV infection by inhibiting S protein in Vero E6 cells - Blocks the S protein-ACE2 interaction in a cell-free competition assay	[18,40,41,46,47]
Alkaloids	Quinine, cinchonine Cepharanthine, fangchinoline, tetradrine Lycorine	Cinchona trees Stephania flowering plants Cultivated bush lily, daffodils	- DNA intercalators, inhibit replication, transcription, and translation - Suppresses inflammatory response in mouse model of lung injury - Inhibit expression of viral spike and nucleocapsid proteins - Inhibits viral replication - Inhibits autophagy in Vero cells	[62,63,64,65]
Cannabinoids	Cannabidiol extracts CBD, CBG, and THC extracts CBD extract	Cannabis Cannabis Cannabis	- Decreased ACE2 and TMPRSS2 protein levels in 3D human oral, airway, and intestinal tissue model - Decrease TNF-α-induced inflammation in lung epithelial cells - Decreases infiltrating neutrophils and cytokine levels in Poly(I:C)-induced sings of ARDS	[66,67,68]
Plant lipids	Omega 3 fatty acid	Variety of foods	- Increased survival rate and decreased BUN, Cr, and K levels in COVID-19 patients	[69]
Prebiotic polysaccharides	Fiber	Whole wheat	- Promote immunity by bacterial translocation across gut’s wall - Reduces inflammatory incidence - Antioxidant	[70,71]

### 3.2. Alkaloids

Alkaloids are naturally occurring compounds that contain at least one nitrogen as a heteroatom in a heterocyclic ring, which is essential for producing the physiological response. Alkaloids are classified into (1) true alkaloids (derived from amino acids and contain a nitrogen atom), (2) proto-alkaloids (derived from amino acids but do not contain a nitrogen atom), and (3) pseudoalkaloids (not derived from amino acids). Intercalating alkaloids, such as resochin, palmatine, and chelidonine, are also considered potential drug candidates because of their antiviral properties and ability to hinder replication, transcription, and translation of the viral genome [63].

Isoquinoline alkaloids, such as ß-carboline, and quinoline alkaloids, such as quinine, cinchonine, dictamine, and skimmianine, are considered as DNA intercalators and have the ability to stabilize double-stranded nucleic acids and inhibit replication, transcription, and translation of genetic material [62]. Being DNA intercalators, these alkaloids have the potential to hinder viral reproduction in cells infected with SARS-CoV-1 and other viruses. Multiple bis-benzylisoquinoline alkaloids, including cepharanthine (CEP), fangchinoline (FAN), and tetrandrine (TET) (Figure 5) have been tested in the suppression of human coronavirus infection. These compounds exhibit both anti-inflammatory and anticancer properties, allowing them to aid in controlling outcomes caused by cytokine storms [63]. These alkaloids have also proven beneficial in inhibiting the expression of viral spike and nucleocapsid protein. Lycorine, derived from *Lycoris radiate*, a traditional Chinese medicinal herb, possesses antiviral and anti-inflammatory properties. Prior study in an in vitro viral replication model demonstrated that lycorine inhibits SARS-CoV2 likely by (1) blocking the elongation of viral RNA translation and suppressing viral RNA replication [64], and (2) negatively targeting autophagy in human enterovirus 71 (EV71)- and Coxsackievirus A16 (CVA16)-infected African green monkey kidney (Vero) cells [65]. Furthermore, in silico reports demonstrated suitable binding affinity of lycorine to 3CL^pro^ of coronaviruses, especially the SARS-CoV-2 [72], which suggests that in vivo experiments are merited (Table 1).

Hydroxychloroquine (HCQ) and chloroquine (CQ) are molecular compounds synthetically derived from quinine, an alkaloid extract from the tree bark of *Remija* and *Cinchona* (Rubiaceae) [73]. In 2014, the FDA approved four small molecules against MERS-CoV, including CQ, chlorpromazine (rauwolfia alkaloid from reserpine), loperamide, and lopinavir (ergot alkaloid), which all inhibit MERS-CoV replication in micromolar concentrations [74]. However, no drugs or biologics have been approved by the FDA for the treatment of COVID-19. HCQ and CQ are well-known antimalarial drugs and have been tested against human coronavirus since the outbreak of COVID-19. A prior study indicated that CQ phosphate inhibits phosphorylation of ACE2 in Vero E6 non-human primate cells [4]. Another study suggested that CQ and HCQ suppress virus replication by increasing endosomal pH, which is crucial for viral replication [75,76]. A considerable number of clinical trials were initiated in China, Italy, Spain, Great Britain, and Thailand to test the therapeutic efficacy of CQ and HCQ against COVID-19 (NCT04303507 and NCT04303299) [77,78].

Cepharanthine (CEP, Figure 5) is a naturally occurring alkaloid derived from *Stephania cepharantha.* CEP demonstrates anti-inflammatory, antioxidative, immunomodulating, antiparasitic, and antiviral properties. Notably, the anti-inflammatory properties were tested in an in vivo mouse model of mastitis. Mice treated with CEP suppress inflammatory response by reducing the levels of TNFα, IL-1β, and IL-6 [79], suggesting CEP may be useful for controlling the cytokine storm associated with COVID-19. CEP also suppresses the inflammatory response and inhibits vascular smooth muscle cell proliferation and migration during atherosclerosis by repressing NF-κB, lipid peroxidation, NO production, and expression of cyclooxygenase in both a mouse model of acute lung injury and LPS-stimulated RAW264.7 cells [80]. In addition, several in vitro studies have noted the antiviral properties of CEP against a variety of viruses, such as HIV, human T-lymphocytic virus (HTLV), HBV, SARS-CoV, and HCoV-OC43 [81].

Importantly, several clinical trials are ongoing on alkaloids, such as colchicine (NCT04527562, NCT04392141, NCT04375202, NCT04355143, and NCT04360980), berberine (NCT04479202), and tetrandrine (NCT04308317). Many alkaloids have exhibited high efficacy as anti-SARS-CoV-2 agents. Together, these in vivo, in vitro, and clinical trial studies indicate that alkaloids can be the potential drug of choice in managing complications associated with COVID-19.

### 3.3. Cannabinoids

The cannabis plant, *Cannabis sativa,* contains more than 600 chemical constituents, and among them are approximately 150 cannabinoids. Some of the main pharmacologically active compounds include psychoactive tetrahydrocannabinols (THC), ∆8-THC, and ∆9-THC. Non-psychoactive cannabinoids include cannabinol (CBN) and cannabidiol (CBD) (Figure 5), and non-cannabinoids include flavonoids, terpenes, and fatty acids [82]. Endocannabinoids (eCBs) include a group of physiological endogenous lipid mediators, including N-arachidonoylethanolamine (anandamide, AEA) and 2-arachidonoylglycerol (2-AG). Depending on the physiological and pathological stimuli, 2-AG and AEA are synthesized and released into many cells and tissues. Both 2-AG and AEA are oxidized into prostanglandin-ethanolamines, prostaglandin-glyceryl esters, hydroxyl-anandamides, and hydroxyeicosatetraenloyl-glycerol [83,84]. Both eCBs have been implicated in the regulation of the immune system and have various beneficial effects against several chronic inflammatory diseases. Furthermore, eCBs also exhibit antiviral effects against HIV [85], viral hepatitis [86], and influenza [83], suggesting a possibility for considering cannabinoids as a treatment option in COVID-19 patients. Wang et al. analyzed the effects of 23 cannabis extracts on ACE2 expression by using 3D human oral, airway, and intestinal tissue models [66]. They found that 13 high CBD extracts decreased ACE2 and TMPRSS2 protein levels, which are crucial for SARS-CoV-2 virus entry into host cells [66]. In a separate study, the authors extracted CBD, CBG, and THC and examined their activity in a model of TNFα-induced inflammation in lung epithelial cancer cells, A549 [67]. All three extracts reduced IL-6, IL-8, CCL2/7, and ACE2 expression and induced macrophage polarization and phagocytosis in differentiated KG1 cells. Interestingly, in a recent study, mice were given a synthetic analog of viral double-stranded RNA known as polyinosinic:polycytidylic acid (Poly (I:C)) via intranasal administration to simulate SARS-CoV-2-mediated signs of ARDS and cytokine storm [68]. CBD treatment downregulated the number of infiltrating neutrophils and macrophages and significantly reduced cytokine levels (IL-6, TNFα, and INF-γ) in the lungs of the mice [68] (Table 1). Importantly, there is an ongoing clinical trial to assess the efficacy and safety of CBD (300 mg/day) in patients infected with SARS-CoV-2. The objective of this study is to examine whether CBD administration in patients decreases viral load, modifies the inflammatory response, reduces clinical and emotional symptoms, and reduces hospitalization and disease severity (NCT04467918). Together, these findings suggest a potential protective role of cannabinoids to reduce local or systemic inflammation in COVID-19.

### 3.4. Plant Lipids

Linoleic acid (LA) is one of the polyunsaturated fatty acids (PUFA) and is considered essential as it cannot be synthesized in animals and humans and is found in most western diets. The dietary sources of LA include vegetable oils, nuts, seeds, and margarine butter. LA is metabolized to form gamma-linolenic acid (GLA), elongated to dihomo-gamma-linolenic acid (DGLA), and then desaturated to form arachidonic acid (ARA) [87]. ARA is cleaved at the sn-2 position by phospholipase A2, which is activated by many inflammatory stimuli. The released ARA then serves as a substrate for cyclooxygenase-2 (COX-2), 5-lipoxygenase, and thromboxane synthase enzymes to form eicosanoids, such as prostaglandins (PG), leukotrienes (LT), and thromboxane (TX). These compounds act as mediators and regulators of inflammatory processes. Prostaglandin E2 (PGE2) and LTB4 are proinflammatory in nature, where activation of PGE2 induces fever, increases vasodilation and vascular permeability, activates pain perception, and proinflammatory cytokine IL-6. On the other hand, omega-3 PUFAs, which include eicosapentaenoic acid (EPA) and docosahexaenoic acid (DHA) (Figure 5), provide protection against inflammation. Interestingly, both EPA and DHA competitively inhibit and replace ARA at the cell membrane phospholipid, shifting the pathway away from proinflammatory and towards EPA- and DHA-mediated amplification of anti-inflammatory response.

A randomized, double-blind placebo-controlled clinical trial in aging adults with at least one known chronic inflammatory nonautoimmune condition was performed to determine the effect of EPA and DHA supplementation on inflammatory biomarkers. The study demonstrated that EPA and DHA supplementation for four weeks significantly lowered the plasma cytokine levels of IL-6, IL1β, and TNFα, whereas supplementation for eight weeks resulted in an even greater reduction in inflammatory cytokines [88]. This finding was consistent with other in vitro studies [89,90] and randomized clinical trials [91,92]. Recently, a double-blind, randomized clinical trial was performed to examine the effect of EPA and DHA on inflammatory and biochemical markers in critically ill COVID-19 patients. This study demonstrated an increase in survival rate, increase in pH and HCO_3,_ and decrease in BUN, Cr, K and levels compared to the control group [69]. Interestingly, despite the fact that the focus of this clinical trial was to examine the effect of omega-3 FAs on inflammatory biomarkers, the author did not have enough resources to estimate inflammatory biomarkers in COVID-19 patients [69]. However, considering the anti-inflammatory effects of omega-3 FAs demonstrated in previous in vitro studies and clinical trials, omega-3 FAs supplementation is likely to be beneficial in reducing systemic inflammation in COVID-19 patients.

### 3.5. Prebiotic Polysaccharides (Fibers)

Fibers can be classified as a macronutrient, encompassing carbohydrates and carbohydrate-containing compounds that cannot be digested or absorbed by the small intestine [93]. Fibers are primarily found in fruits and vegetables and play an important role in maintaining gut health [71]. Fibers have been shown to modulate the immune system, both directly and indirectly, by modifying the composition of the gut microbiota and their production of short-chain fatty acids (SCFAs) [94].

As discussed above, ACE2 receptors play a vital role in the spread of SARS-like coronaviruses. It has been observed that ACE2 mutations change the gut microbial composition, which leads to gastrointestinal dysfunction in patients suffering from COVID-19 [70]. Moreover, viral infection is also associated with defects in intestinal epithelial barrier integrity, immune response, and gut microbiota balance, resulting in activation of the immune response, an increase in inflammation, and, ultimately, disease progression. A high-fiber diet helps preserve a healthy gut microbial environment by reducing inflammation, which aids in strengthening the immune system in patients with COVID-19 infection [70].

Similarly, patients with virus-mediated (influenza A virus, respiratory syncytial virus, and recombinant pneumonia virus) respiratory infection have also experienced dysbiosis and subsequent dysregulation in immunological processes that have been linked to the microbiome [95,96,97]. Imbalances in gut microbiota due to smoking, use of antibiotic, or some types of diets lead to inflammation in the gut and likely predispose the distal organs to microbial infection, such as lung [98]. Reciprocally, the lung microbiota likely causes an imbalance in gut microbiota composition in response to influenza infection. Therefore, the “gut–lung axis”, also referred to as gut microbiota–lung immunity, is crucial during respiratory diseases mediated by viral infection. Prior studies have also suggested that the mechanism responsible for the imbalance in the gut microbiome during respiratory infection is likely by induction of type I interferons (IFNs) [99]. Indeed, it is known that patients with SARS-CoV-2 infection suffering from ARDS also exhibit gastrointestinal symptoms, such as diarrhea, vomiting, nausea, and abdominal pain [100], which may be related to similar mechanisms.

## 4. Summary and Conclusions

In this review, mechanisms of SARS-CoV-2 infection, including adhesion, entry, and replication into host cells, have been outlined to design potential treatment options. The antiviral and anti-inflammatory effectiveness of plant-derived bioactive compounds has been previously described in HIV, HSP, influenza, and MERS. With the ongoing COVID-19 pandemic, plant-derived bioactive compounds have gained attention for their possible use in mitigating SARS-CoV-2 infection. The majority of existing studies have tested the efficacy of these compounds using in silico computational models or in vitro cell culture models. However, there is still a lack of in vivo studies to demonstrate the effects of plant-derived bioactive compounds against COVID-19 infection. Several clinical studies are ongoing to assess the pharmacological potential of these compounds for managing symptoms associated with COVID-19 infection. Furthermore, clinical studies demonstrating the relationship between dietary aspects of plant-derived compounds and prevention of complications of COVID-19 infections will provide valuable information in recommending bioactive compounds as nutritional supplements and their applications as nutraceuticals against SARS-CoV-2 infection.

## Data Availability

Not applicable.
